# Coordinated Development of Immune Cell Populations in Vascularized Skin Organoids from Human Induced Pluripotent Stem Cells

**DOI:** 10.1002/adhm.202502108

**Published:** 2025-08-16

**Authors:** Mitchell Mostina, Jane Sun, Seen Ling Sim, Imaan A. Ahmed, Fernando Souza‐Fonesca‐Guimaraes, Ernst J. Wolvetang, Jason Brown, Snehlata Kumari, Kiarash Khosrotehrani, Abbas Shafiee

**Affiliations:** ^1^ Experimental Dermatology Group Dermatology Research Centre The University of Queensland Frazer Institute, The University of Queensland Brisbane QLD 4102 Australia; ^2^ The University of Queensland Frazer Institute The University of Queensland Woolloongabba Brisbane QLD 4102 Australia; ^3^ Australian Institute for Bioengineering and Nanotechnology The University of Queensland Brisbane QLD 4072 Australia; ^4^ Royal Brisbane and Women's Hospital Metro North Hospital and Health Service Queensland Health Brisbane QLD 4029 Australia; ^5^ Herston Biofabrication Institute Metro North Hospital and Health Service Queensland Health Brisbane QLD 4029 Australia; ^6^ Department of Dermatology Princess Alexandra Hospital Metro South Hospital and Health Service Queensland Health Brisbane QLD 4102 Australia

**Keywords:** biofabrication, disease modeling, hair follicle, regenerative medicine, wound healing

## Abstract

Human skin is a highly vascularized organ, where blood vessels perform essential roles in maintaining skin homeostasis and participate in thermal regulation. Currently, there is an unmet challenge to generate vascularized, functional skin models. In this study, human fetal placental endothelial colony forming cells (ECFC) are incorporated into human induced pluripotent stem cell (hiPSC)‐derived skin organoids (SKO), forming capillary‐like structures and generating endothelialized SKOs. However, this approach is limited by the inability of ECFCs to establish a complete vascular network, and impeding full epidermal stratification and hair follicle morphogenesis. In independent experiments, hiPSC‐derived vascular organoids (VO), are incorporated into the SKO, forming complex vascular structures and generating fully vascularized skin organoids (VSKO) with resident immune cell populations. Immunofluorescence microscopy and flow cytometric analyses reveal the transfer and integration of endothelial, mural, hematopoietic, and mesenchymal cells from VOs into the skin components of VSKOs. This study pioneers the establishment of VSKOs as a transformative platform for studying human skin biology, and immune‐skin interactions with applications in investigating inflammatory and other immune‐mediated skin disorders.

## Graphical abstract

Vascularized skin organoids are developed from human induced pluripotent stem cells and contain macrophages, Langerhans cells, and neutrophils. Vascularized skin organoids offer a transformative platform to model skin biology and enable mechanistic investigations of inflammatory and hematologic skin disorders. Immunofluorescence microscopy image of vascularized skin organoids were stained with cytokeratin 10 (yellow), and platelet‐derived growth factor receptor α (magenta). Green color represents green fluorescent protein‐tagged vasculature.

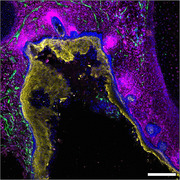

## 1 Introduction

Known as the largest organ of the human body, skin exerts many vital functions.^[^ ^1^ ^]^ Acting as a barrier to the external environment, skin protects internal organs from invading microorganisms, mechanical and chemical trauma, ultraviolet radiation, and avoids the loss of water and heat.^[^ ^1^ ^]^ Notably, the skin has three key layers: the epidermis, dermis, and a hypodermal subcutaneous layer of adipose tissue. In the reticular region of the dermis, dense connective tissue and bundles of collagen fibers facilitate the formation of skin appendages, including hair follicles and blood vessels.^[^ ^1^, ^2^ ^]^ Furthermore, hair follicles and blood vessels extend and maintain their homeostatic functions to the hypodermal region.^[^ ^1^, ^2^ ^]^

Hair follicles are important components of the integument, undergoing cyclic phases of anagen (growth), catagen (transition), and telogen (rest).^[^ ^3^ ^]^ In addition, regulatory T cells and specific macrophage subsets localize near the hair follicle bulge and regulate the hair follicle cycle, highlighting an essential homeostatic immune interaction.^[^ ^4^, ^5^, ^6^, ^7^ ^]^ It was suggested that hair follicles serve as entry points for Langerhans cell precursors into the epidermis, contributing to innate immunity.^[^ ^7^, ^8^ ^]^ Furthermore, hair follicles facilitate dendritic cells and T cell trafficking, acting as essential sites for cutaneous antigen presentation.^[^ ^7^, ^9^ ^]^

The dermal vasculature enables metabolic exchange and nutrient perfusion to the epidermis.^[^ ^10^ ^]^ Insufficient blood supply to the skin can lead to tissue necrosis, infection, and sepsis, highlighting the immunological importance of skin vasculature.^[^ ^11^, ^12^ ^]^ During embryogenesis, blood vessels form through *de novo* vasculogenesis, driven by specific transcription factors and signaling‐mediated differentiation of angioblasts into endothelial cells.^[^ ^13^, ^14^, ^15^, ^16^, ^17^ ^]^ The main driver of this process is vascular endothelial growth factor (VEGF) which promotes vasculogenesis and angiogenesis in coordination with Notch signaling.^[^ ^18^, ^19^ ^]^

Endothelial cells are surrounded by a basement membrane and pericytes to support the structural integrity of blood vessels.^[^ ^8^ ^]^ The “perivascular extravasation units” are critical in enabling neutrophil extravasation for migration to sites of foreign microbial invaders.^[^ ^8^ ^]^ Furthermore, perivascular macrophages express high levels of neutrophil‐attracting chemokines such as C‐X‐C motif chemokine ligand (CXCL)1 and CXCL2, to support this extravasation process.^[^ ^8^ ^]^ In the absence of perivascular macrophages, neutrophil recruitment to the skin is suppressed.^[^ ^8^ ^]^ Also, T cell recruitment is crucially influenced by postcapillary venules.^[^ ^20^ ^]^ During cutaneous inflammation, T cells, dendritic cells, and perivascular macrophages form clusters around the postcapillary venules.^[^ ^20^ ^]^ This is known as the inducible skin‐associated lymphoid tissue, where these clusters act as a site of antigen presentation in the skin to promote an adaptive immune response.^[^ ^20^ ^]^ Overall, vasculature has an essential role in many aspects of skin physiology.

Human skin includes a vast variety of cell types and intricate immune‐hair follicle interactions, making it challenging to accurately model in vitro. While many attempts to generate complex 3D skin equivalents have achieved vascularization, hair follicle generation, and immune cell co‐cultures, there has been a complete absence of endogenously differentiating a vascularized, hair‐bearing human skin equivalent that contains immune cell populations.^[^ ^21^, ^22^, ^23^, ^24^, ^25^, ^26^, ^27^ ^]^

Recent advances in human induced pluripotent stem cell (hiPSC) technology have enabled the generation of skin organoids (SKO) with hair follicles, pigmentation, dermal stratification, appendage patterning, and innervation.^[^ ^25^, ^26^, ^28^, ^29^, ^30^, ^31^, ^32^, ^33^, ^34^, ^35^ ^]^ Despite their experimental advantages over existing skin equivalents, the physiological relevance of hiPSC‐derived SKOs remains limited by the absence of vascular and immune components, as SKOs are derived from differentiation of the surface ectoderm.^[^ ^36^, ^37^, ^38^ ^]^ hiPSCs offer a unique opportunity to generate cell types from different germ layers which can then be assembled to create more complex and physiologically relevant skin substitutes.^[^ ^39^, ^40^ ^]^

The therapeutic efficacy of skin substitutes is limited due to their inadequate vascularization, leading to poor graft integration and increased risk of infection. To address these limitations, research has progressed toward the development of pre‐vascularized skin substitutes that support functional engraftment.^[^ ^41^ ^]^ The use of pre‐vascularized skin substitutes allows efficient blood supply and effective tissue skin regeneration.^[^ ^41^ ^]^ Thus, endothelial progenitor cells and vascularized skin substitutes have been explored to enhance wound healing outcomes.^[^ ^42^, ^43^, ^44^, ^45^ ^]^ Endothelial colony forming cells (ECFC), sourced from cord‐blood or the placenta, demonstrate the ability to create *de novo* vascular networks in vitro, whilst maintaining the capacity to integrate with host vasculature in vivo.^[^ ^46^, ^47^, ^48^ ^]^ Similarly, ECFCs and endothelial cells can be sourced from hiPSCs.^[^ ^49^, ^50^ ^]^ More recently, to provide the full range of cells composing blood vessels, hiPSC‐derived vascular organoids (VO) have been developed.^[^ ^51^ ^]^ Vascular progenitors originate from the mesoderm, where they can contribute to *de novo* vasculogenesis and support angiogenesis.^[^ ^52^ ^]^ This enables recapitulation of not only endothelial cells, but the structurally supporting mural cell types such as pericytes, and vascular smooth muscle cells (VSMC).

Various vascular cell sources have been explored to vascularize hiPSC‐derived organoids, such as brain organoids.^[^ ^53^, ^54^ ^]^ Although macrophage co‐culture with SKOs has been reported,^[^ ^35^ ^]^ a physiologically relevant vascularized hiPSC‐derived SKO has yet to be achieved.^[^ ^36^, ^37^, ^38^ ^]^ This study addresses this gap in hiPSC‐derived SKOs, by coordinating the differentiation of vascular and skin lineages to generate immune‐integrated vascularized skin organoid (VSKO).

## 2 Results

### 2.1 Skin Organoids Can Be Endothelialized

**Figure 1:**
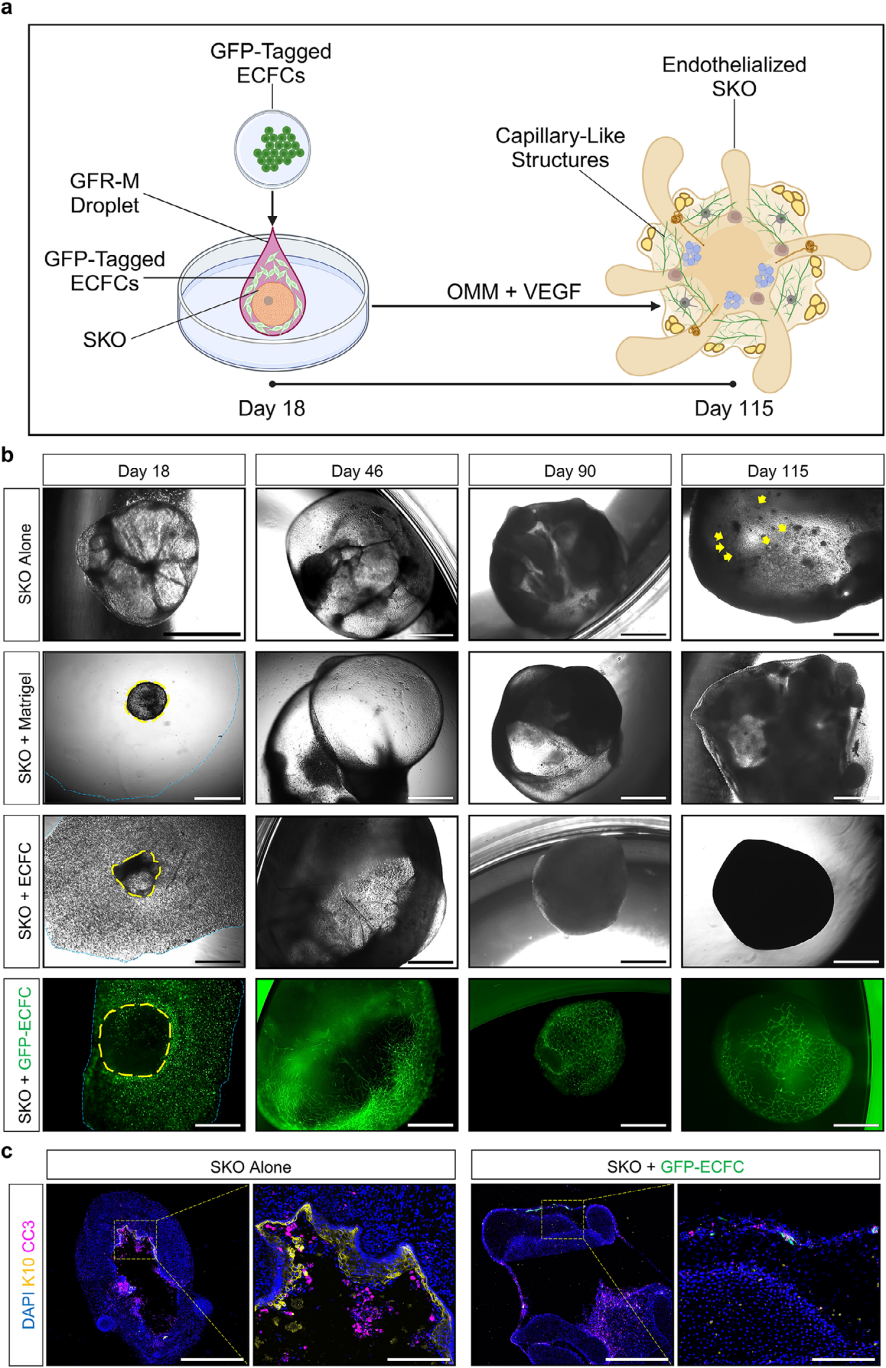
Development of Endothelialized Skin Organoids. a) Overview of the co‐culture of one skin organoid (SKO) with green fluorescent protein (GFP)‐tagged endothelial colony forming cells (ECFC). Created with BioRender.com. b) Representative brightfield microscopy images of P111 human induced pluripotent stem cell (hiPSC)‐derived SKOs with all experimental conditions at day 18, 46, 90, and 115 of SKO differentiation. Scale bars are 1 mm. Yellow colored arrows indicate hair follicles. Yellow color circles indicate SKOs. Blue color circles indicate the Growth Factor Reduced Basement Membrane Matrigel® Matrix (GFR‐M) droplet. c) Representative immunofluorescence microscopy images of P111 hiPSC‐derived SKOs at day 115 of differentiation stained with cleaved caspase‐3 (CC3, magenta color) and cytokeratin 10 (K10, yellow color) antibodies. Scale bars are 1 mm. Magnified image scale bars are 500 µm. Yellow color boxes indicate regions of interest. Cell nuclei are stained with 4′,6‐diamidino‐2‐phenylindole (DAPI, blue color). Organoid maturation medium (OMM). Vascular endothelial growth factor (VEGF).

SKOs were generated from hiPSCs (Figure S1, Supporting Information). Prior to co‐culture, green fluorescent protein (GFP)‐tagged ECFCs were cultured in EGM‐2 Endothelial Cell Growth Medium‐2 Bulletkit (EGM2) supplemented with an increasing percentage of organoid maturation medium (OMM) containing 5 ng mL^−1^ VEGF, to prime them for co‐culture. Considering vasculogenesis occurs in human embryos on day 18, we recapitulated the process of human skin development and co‐cultured the primed ECFCs with SKOs at day 18 of SKO differentiation (**Figure** **1**a). The co‐culture was maintained in OMM with VEGF until day 115, to promote vascularization (Figure 1a,b). Control groups were also established (Figure 1b).

By day 46, the GFP^+^ endothelial capillaries surrounded SKOs entirely, as the Growth Factor Reduced Basement Membrane Matrigel Matrix (GFR‐M) droplet degraded (Figure 1b). The presence of endothelial capillaries was confirmed for the entire duration of the experiment.

At day 115 of SKO differentiation, SKOs were stained with cleaved caspase‐3 (CC3) and cytokeratin 10 (K10) to assess potential formation of a necrotic core within the organoids and keratinocyte stratification, respectively. Immunofluorescence microscopy confirmed the absence of necrotic cores in both SKO and SKO + GFP‐ECFC models at day 115 of SKO differentiation (Figure 1c). CC3^+^ cells within the SKO Alone group may indicate corneocyte maturation and their shedding from the epithelial layers (K10^+^) into the organoid's cyst. Notably, K10 analysis confirmed the absence of K10^+^ cells in the SKO + GFP‐ECFC group, highlighting the inhibitory effects of ECFCs and GFR‐M on SKO maturation.

### 2.2 Endothelialized Skin Organoids Recapitulate Endothelial Structures in Vitro

**Figure 2:**
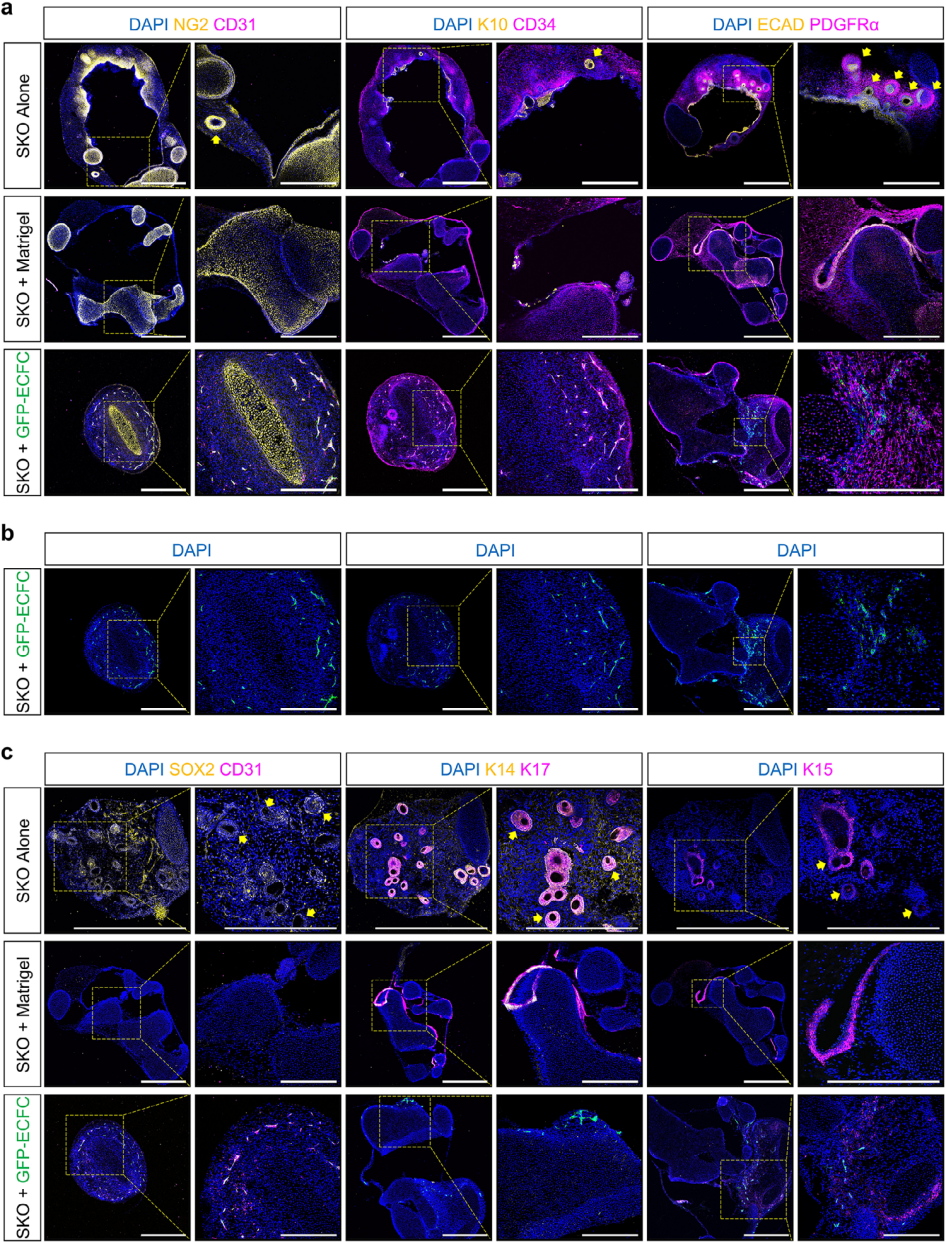
Skin Organoids Contain Endothelial Cells. a) Representative immunofluorescence microscopy images of day 115 P111 human induced pluripotent stem cell (hiPSC)‐derived skin organoids (SKO) with all experimental conditions. The presence of cluster of differentiation (CD)31^+^ and CD34^+^ staining (both magenta color) confirmed the formation of capillary‐like structures in the SKO + green fluorescent protein (GFP)‐endothelial colony formin cells (ECFC) group, which overlapped with GFP^+^ expression. In contrast, no CD31^+^ areas were detected in the SKO Alone or SKO + Matrigel groups. Cytokeratin 10^+^ (K10, yellow color) staining demonstrated the maturation of ketatinocytes in both the SKO Alone and SKO + Matrigel groups, further confirmed by positive expression of epithelial cadherin (ECAD, yellow color) in the epithelial layer. Notably, hair follicles are present only in the SKO Alone group, and stained positively for platelet‐derived growth factor receptor α (PDGFRα, magenta color). Immunofluorescence microscopy of neural/glial antigen 2 (NG2), a key marker for mesenchymal cells and perciytes, revealed abundant cartilage, as well as NG2^+^ capillary‐like structures in the SKO + GFP‐ECFC group. Scale bars are 1 mm. Magnified image scale bars are 500 µm. b) Representative immunoflourescence microscopy images of day 115 P111 SKO + GFP‐ECFC condition with the GFP channel only, confirms the presence of GFP‐tagged ECFCs around the SKO. Scale bars are 1 mm. Magnified image scale bars are 500 µm. c) Representative immunoflourescence microscopy images of all experimental conditions at day 115 of SKO differentiation. SRY‐box transcription factor 2^+^ (SOX2, yellow color) cells, a key marker for hair follicles, were detected only in the SKO Alone group. The presence of hair follicles were further confirmed by the co‐expression of K14 and cytokeratin 17 (K17). Cytokeratin 15 (K15) and K17 expression were observed in both the hair follicle regions and epithelial layer of the SKO Alone and SKO + Matrigel groups, suggestive of keratinization in these experimental groups. No SOX2^+^, K14^+^, K15^+^, or K17^+^ cells were detected in the basal or suprabasal layer of SKO + GFP‐ECFC group, indicating the inhibitory effect of ECFCs on hair follicle development in this setting. Scale bars are 1 mm. Magnified image scale bars are 500 µm. Cell nuclei are stained with 4′,6‐diamidino‐2‐phenylindole (DAPI, blue color). Yellow colored arrows indicate hair follicles. Yellow color boxes indicate regions of interest.

Endothelialized SKOs were characterized by immunofluorescence microscopy (**Figure** **2**a–c). We observed that the SKOs had no discernible impact on ECFC survival (Figure 2a–c). Furthermore, the customized OMM supported the co‐existence of both ECFCs and SKOs for the experiment's entire duration, up to day 115 of differentiation (Figure 2a–c). However, compared to the SKO + Matrigel control group, the GFR‐M droplet containing ECFCs impacted the cyst‐like structure of the SKO, where heterogeneity was observed with some endothelialized SKOs containing a neural/glial antigen 2 (NG2)^+^ cartilaginous structure in the center (Figure 2a). Interestingly, unlike the SKO Alone group, SKO + Matrigel and SKO + GFP‐ECFC groups did not demonstrate hair follicle development (Figure 2c). Additionally, the SKO + GFP‐ECFC group did not demonstrate appropriate keratinization (Figure 2c).

Comparatively, immunofluorescence microscopy confirmed the presence of cluster of differentiation (CD)34^+^ and CD31^+^/NG2^+^ capillary‐like structures in the SKO + GFP‐ECFC group only (Figure 2a). Consequently, this experiment indicated that the ECFCs were the cause of endothelialization in SKOs (Figure 2b), but the necessary addition of the GFR‐M droplet negatively impacted the differentiation process of cells derived from the surface ectoderm, affecting hair follicle formation (Figure 2c).

### 2.3 Human Induced Pluripotent Stem Cells Generate Vascular Organoids

**Figure 3:**
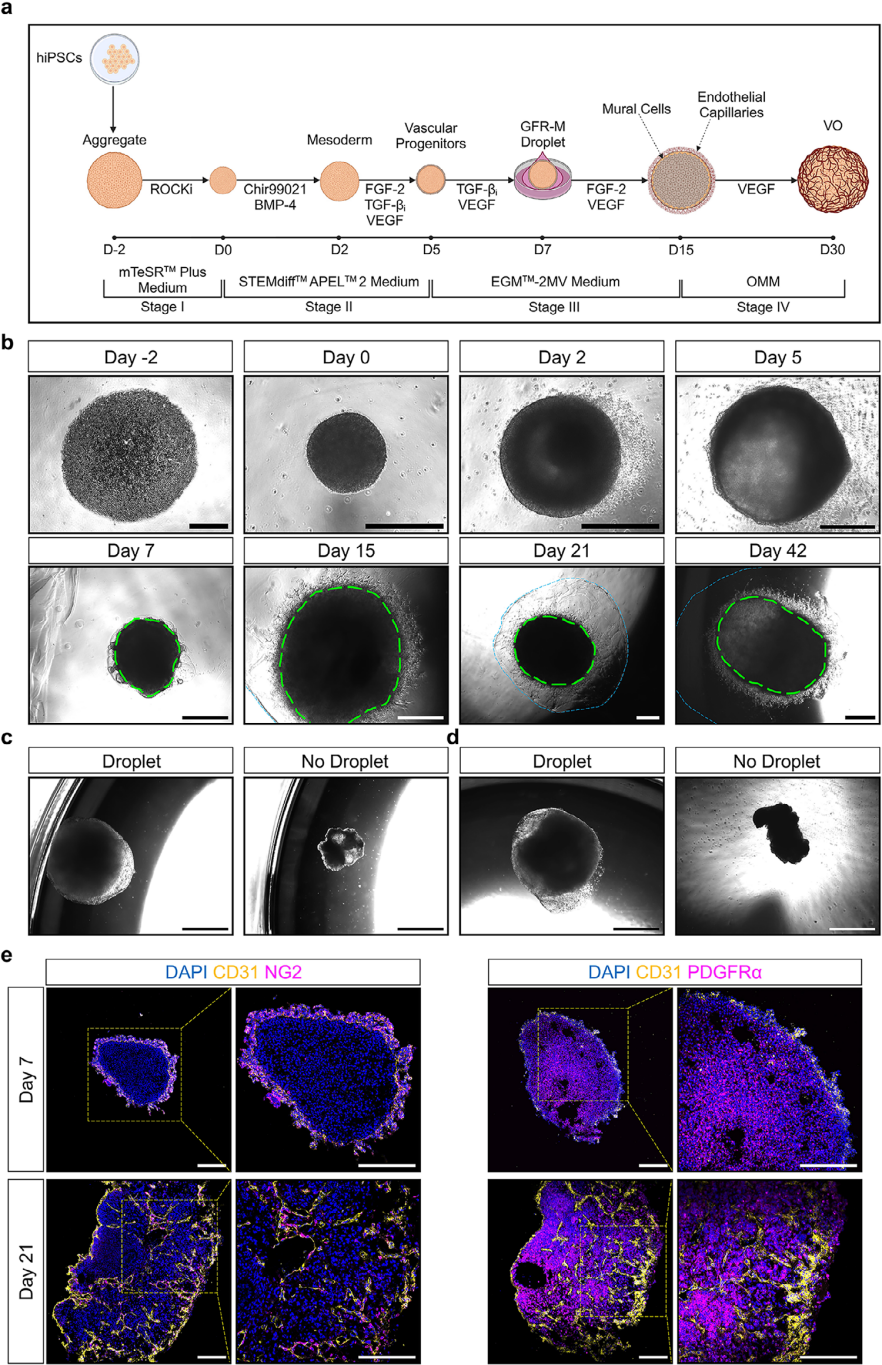
Development of Vascular Organoids. a) Overview of the differentiation protocol of human induced pluripotent stem cells (hiPSC) into vascular organoids (VO). Created with BioRender.com. b) Representative brightfield microscopy images of P111 hiPSC‐derived VOs at key differentiation timepoints. Green color circles indicate VOs. Blue color circles indicate the Growth Factor Reduced Basement Membrane Matrigel® Matrix droplet (GFR‐M) droplet. Scale bars are 500 µm. c) Representative brightfield microscopy images of day 21 P111 hiPSC‐derived VOs with and without GFR‐M droplets. Scale bars are 1 mm. d) Representative brightfield microscopy images of day 21 C32 hiPSC‐derived VOs with and without GFR‐M droplets. Scale bars are 1 mm. e) Representative immunofluorescence microscopy images of day 7 P111 hiPSC‐derived VOs (before the addition of a GFR‐M droplet), and day 21 P111 hiPSC‐derived VOs. While cluster of differentiation (CD)31^+^ (yellow color) and neural/glial antigen 2^+^ (NG2, magenta color) cells are localized at the periphery, the platelet‐derived growth factor receptor α^+^ (PDGFRα, magenta color) cells are present throughout the organoids. Scale bars are 200 µm. Yellow color boxes indicate regions of interest. Cell nuclei are stained with 4′,6‐diamidino‐2‐phenylindole (DAPI, blue color). EGM‐2 MV Microvascular Endothelial Cell Growth Medium‐2 Bulletkit (EGM‐2MV). Bone morphogenetic protein 4 (BMP‐4). Fibroblast growth factor 2 (FGF‐2). Organoid maturation medium (OMM). Transforming growth factor β inhibitor (TGF‐β_i_). StemMACS Y27632 (ROCKi, rho‐associated kinase (ROCK) inhibitor). Vascular endothelial growth factor (VEGF).

Next, we generated VOs by differentiating a 3D aggregate of hiPSCs, similarly to previous studies.^[^ ^51^, ^54^ ^]^ To develop a blood vessel phenotype, we differentiated hiPSCs toward the mesodermal lineage, by adding Chir99021, and bone morphogenetic protein 4 (BMP‐4) at day 0 of VO differentiation, a unique difference with previously described differentiation protocols (**Figure** **3**a). Furthermore, we added transforming growth factor‐β inhibitor (TGFβi) significantly earlier, where on day 7, a GFR‐M droplet was added to the organoids to promote a more prolific and faster endothelial capillary development (Figure 3a).

Brightfield microscopy showed morphological changes of VOs over time as differentiation progressed, including changes in shape, density, and capillary‐like formations (Figure 3b). Compared to the control group, where VOs had no GFR‐M droplet added, VOs with a GFR‐M droplet were able to sustain appropriate differentiation potential, size, and growth (representative brightfield microscopy images of P111 hiPSC‐derived VOs, and C32 hiPSC‐derived VOs, Figure 3c,d, respectively). At day 7, VOs exhibited the presence of CD31^+^ and NG2^+^ cells around the periphery, with platelet‐derived growth factor receptor α (PDGFRα) homogenously expressed throughout the center (Figure 3e).

Also, CD31^+^ and NG2^+^ cells were co‐localized and penetrated toward the center of the VO with the GFR‐M droplet indicating formation of vascular structures (Figure 3e). PDGFRα staining revealed a widespread distribution of positive cells throughout the VOs at day 21 of differentiation (Figure 3e). Our data also highlights the importance of adding a GFR‐M droplet to sustain appropriate differentiation and a heterogenous network of vascular structures that recapitulate blood vessel architecture.

### 2.4 Human Vascular Organoids Recapitulate Broad Blood Vessel Phenotypes

**Figure 4:**
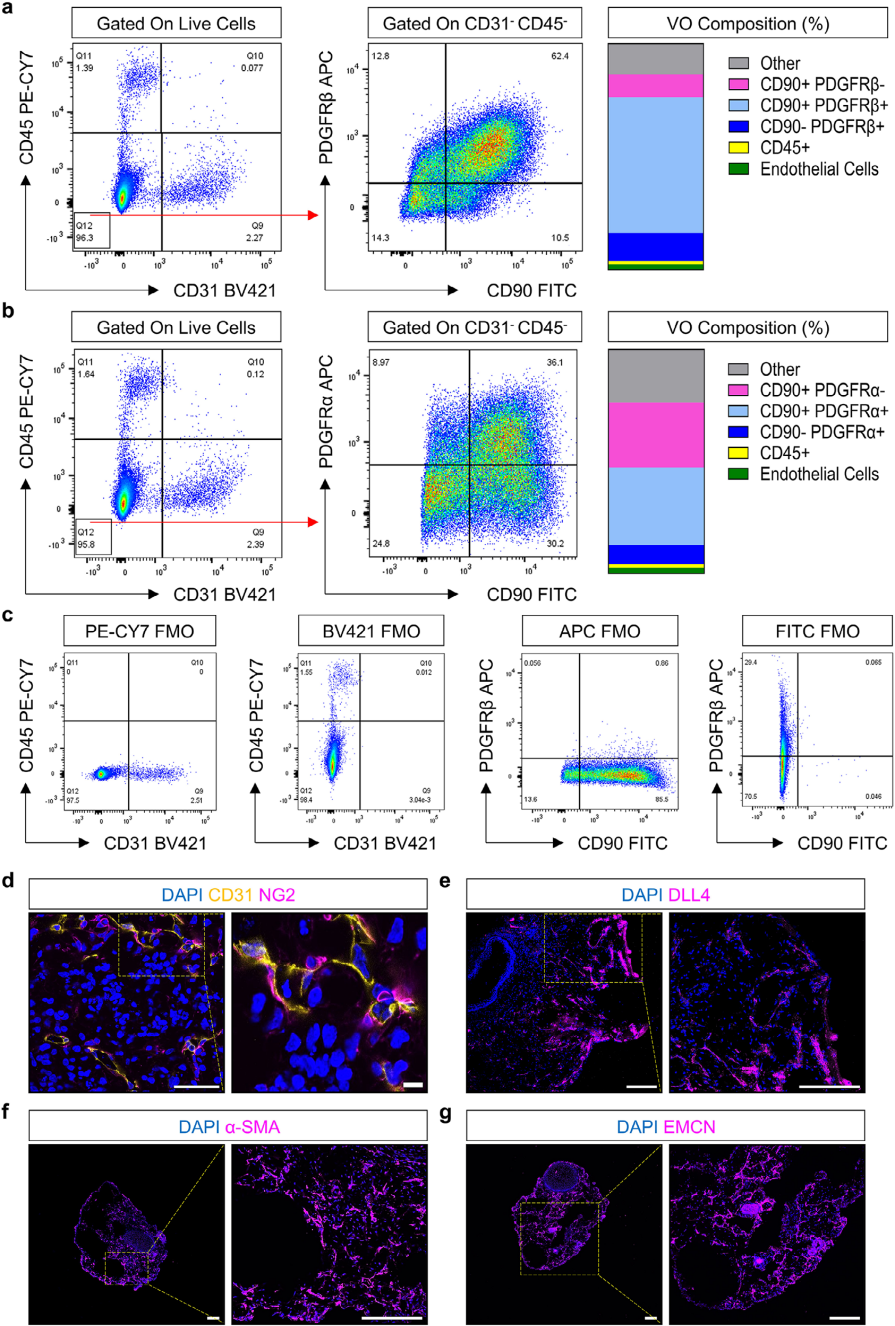
Vascular Organoids Recapitulate a Human Blood Vessel. a, b) Representative flow cytometry plots of day 42 C32 human induced pluripotent stem cell (hiPSC)‐derived vascular organoids (VO). VO are stained with platelet‐derived growth factor receptor β (PDGFRβ) (pericyte marker), PDGFRα, cluster of differentiation (CD)31 (endothelial cell marker), CD45 (hematopoietic cell marker), and CD90 (mesenchymal cell marker) antibodies. The CD31^−^/CD45^−^ population is gated for the expression of PDGFRβ, and PDGFRα markers. Percentage of positive cells (VO Composition %) are determined using fluorescence minus one (FMO) of day 42 C32 hiPSC‐derived VOs c). d) Representative immunofluorescence microscopy images of day 21 P111 hiPSC‐derived VO stained for CD31 (yellow color) and neural/glial antigen 2 (NG2, magenta colour). Scale bar is 50 µm. Magnified image scale bar is 10 µm. e) Representative immunofluorescence microscopy images of day 21 P111 hiPSC‐derived VO demonstrating arterial differentiation with delta‐like protein 4^+^ (DLL4, magenta color) cells. f) Representative immunofluorescence microscopy images of day 21 P111 hiPSC‐derived VO demonstrating α‐smooth muscle actin^+^ (α‐SMA, magenta color) cells. g) Representative immunofluorescence microscopy images of day 21 P111 hiPSC‐derived VO demonstrating venous differentiation with endomucin^+^ (EMCN, magenta color) cells. Cell nuclei are stained with 4′,6‐diamidino‐2‐phenylindole (DAPI, blue color). Scale bars are 200 µm for parts e‐g. Yellow color boxes indicate regions of interest.

To observe the proportion of cell types in the VO, flow cytometric analysis was performed on day 42 of differentiation. VOs demonstrated a heterogenous phenotype of endothelial cells (CD31^+^), hematopoietic cells (CD45^+^), mesenchymal cells (CD90^+^), and pericytes (platelet‐derived growth factor receptor β (PDGFRβ^+^)) (**Figure** **4**a–c). We found most of the VO cells are CD90^+^ cells, which could be PDGFRα^+^ or β+. Figure 4a,b shows that within the CD31^−^/CD45^−^ population, ≈60% were CD90^+^/PDGFRβ^+^, and 36% were CD90^+^/PDGFRα^+^, respectively. This indicates a significant presence of mesenchymal cells within the VOs. In addition, flow cytometric analyses revealed a small proportion of CD45^+^ and CD31^+^ cells. Complementary immunofluorescence microscopy showed that NG2^+^ cells encircled CD31^+^ cells, recapitulating the organizational structure of a human blood vessel (Figure 4d; Video S1, Supplementary Video 1).

Additional immunofluorescence microscopy analyses confirmed the presence of delta‐like protein 4 (DLL4)^+^ arterial capillaries, endomucin (EMCN)^+^ veinous capillaries, and α‐smooth muscle actin (α‐SMA)^+^ mural cells within VOs (Figure 4e–g). This data demonstrates a heterogeneous differentiation of VOs into both arterial and venous lineages and highlights the presence of mature vascular structures. Consequently, this characterization provided sufficient rationale for the use of VOs to appropriately vascularize human skin organoids.

### 2.5 Human Vascular Organoids Contribute to the Vascularization of Skin Organoids

**Figure 5:**
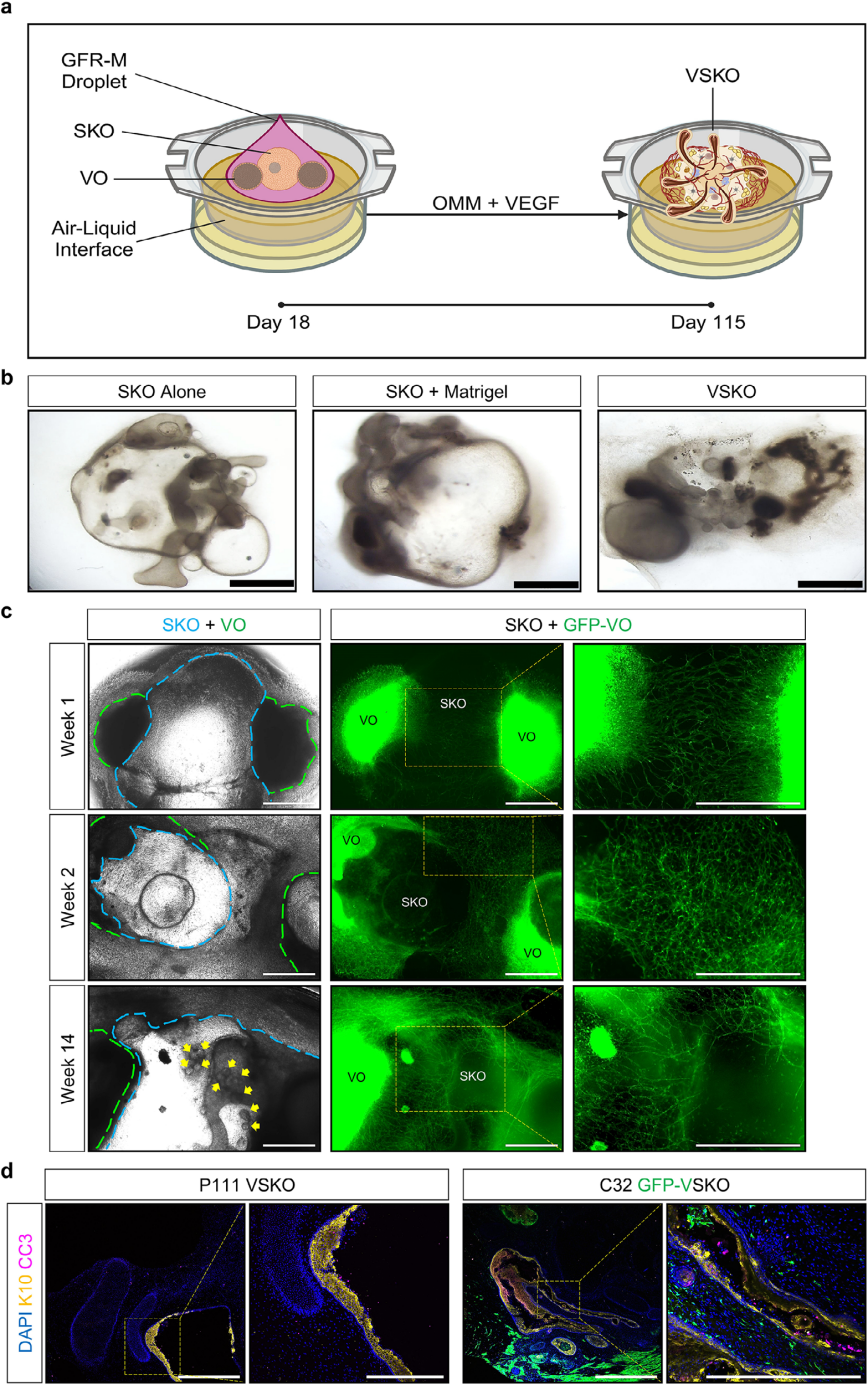
Development of Vascularized Human Skin Organoids. a) Overview of the co‐culture of one skin organoid (SKO) with two vascular organoids (VO) on an air‐liquid interface (ALI). Original figure created with BioRender.com. b) Representative brightfield microscopy images of P111 human induced pluripotent (hiPSC)‐derived SKO Alone and SKO + Matrigel control groups, and the vascularized skin organoid (VSKO) group at day 115 of SKO differentiation. Scale bars are 1 mm. c) Representative brightfield microscopy images of two green fluorescent protein (GFP)‐tagged C32 VOs co‐cultured with one GFP^−^ C32 SKO during week 1, week 2, and week 14 of co‐culture. Blue color circles indicate SKOs. Green color circles indicate VOs. Yellow color arrows indicate hair follicles. Scale bars are 1 mm. d) Representative immunofluorescence microscopy images of day 115 VSKOs‐derived from C32 and P111 hiPSCs stained with cleaved caspase‐3 (CC3, magenta color), and cytokeratin 10 (K10, yellow color) antibodies indicate the absence of necrotic cores, whilst highlighting the formation of a well‐developed, thick epidermal layer within VSKOs. Green color represents GFP^+^ vascular cells migrating from the VO to the skin layers and hair follicles of the VSKO. Scale bars are 1 mm. Magnified image scale bars are 500 µm. Yellow boxes indicate regions of interest. Cell nuclei are stained with 4′,6‐diamidino‐2‐phenylindole (DAPI, blue color). Growth Factor Reduced Basement Membrane Matrigel® Matrix (GFR‐M). Organoid maturation medium (OMM). Vascular endothelial growth factor (VEGF).

To develop vascularized skin in a physiologically relevant environment, we optimized a co‐culture system where from the same hiPSC line, one SKO was placed in between two VOs in a 30 µL GFR‐M droplet, on an air‐liquid interface (ALI) culture using a 24 mm Transwell with 0.4 µm Pore Polyester Membrane Insert (Transwell insert) (**Figure** **5**a).

Exposing the SKO to air recapitulates the external environment human skin is exposed to and has shown to enhance differentiation of hiPSC‐derived SKOs. The ALI system improved SKO development by sustaining hair follicle development and enhanced keratinocyte stratification, when compared to floating culture.^[^ ^33^ ^]^ Figure 5b shows P111 hiPSC‐derived VSKOs, along with SKO Alone and SKO + Matrigel control groups at day 115 of SKO differentiation. The OMM + VEGF medium supported the simultaneous differentiation of both VOs and SKOs over the course of the differentiation period. These findings highlight the effectiveness of the VSKO differentiation protocol for promoting multi‐lineage organoid differentiation and recapitulating the dynamic interplay between vascular and skin tissues. Our data was further confirmed when two GFP‐tagged C32 VOs were co‐cultured with one C32 SKO (GFP^−^) during week 1, week 2, and week 14 of co‐culture (Figure 5c). Notably, the vascular sprouting structures migrated from the VOs toward the SKO.

To demonstrate the reproducibility of this protocol, two hiPSC lines named P111 and C32 were tested. For both C32 and P111 hiPSCs lines, the VSKOs did not demonstrate any necrotic cores after 115 days of SKO differentiation as confirmed by the absence of CC3^+^ cells (Figure 5d). VSKOs showed a well‐developed, thick epidermal layer, as indicated by K10^+^ staining. Additionally, the GFP‐tagged cells from the VOs migrated into the VSKO dermis. The epidermal layers identified as epithelial cadherin (ECAD)^+^ were shown to undergo adequate differentiation as demonstrated by K10 expression. Similarly, GFP^+^ vascular structures could be observed in the dermis and not in the epidermis, demonstrating a successful vascular integration of vessels emanating from the VO to all skin components (Figure 5d).

### 2.6 Vascularized Human Skin Organoids Recapitulate Vascularized Skin

**Figure 6:**
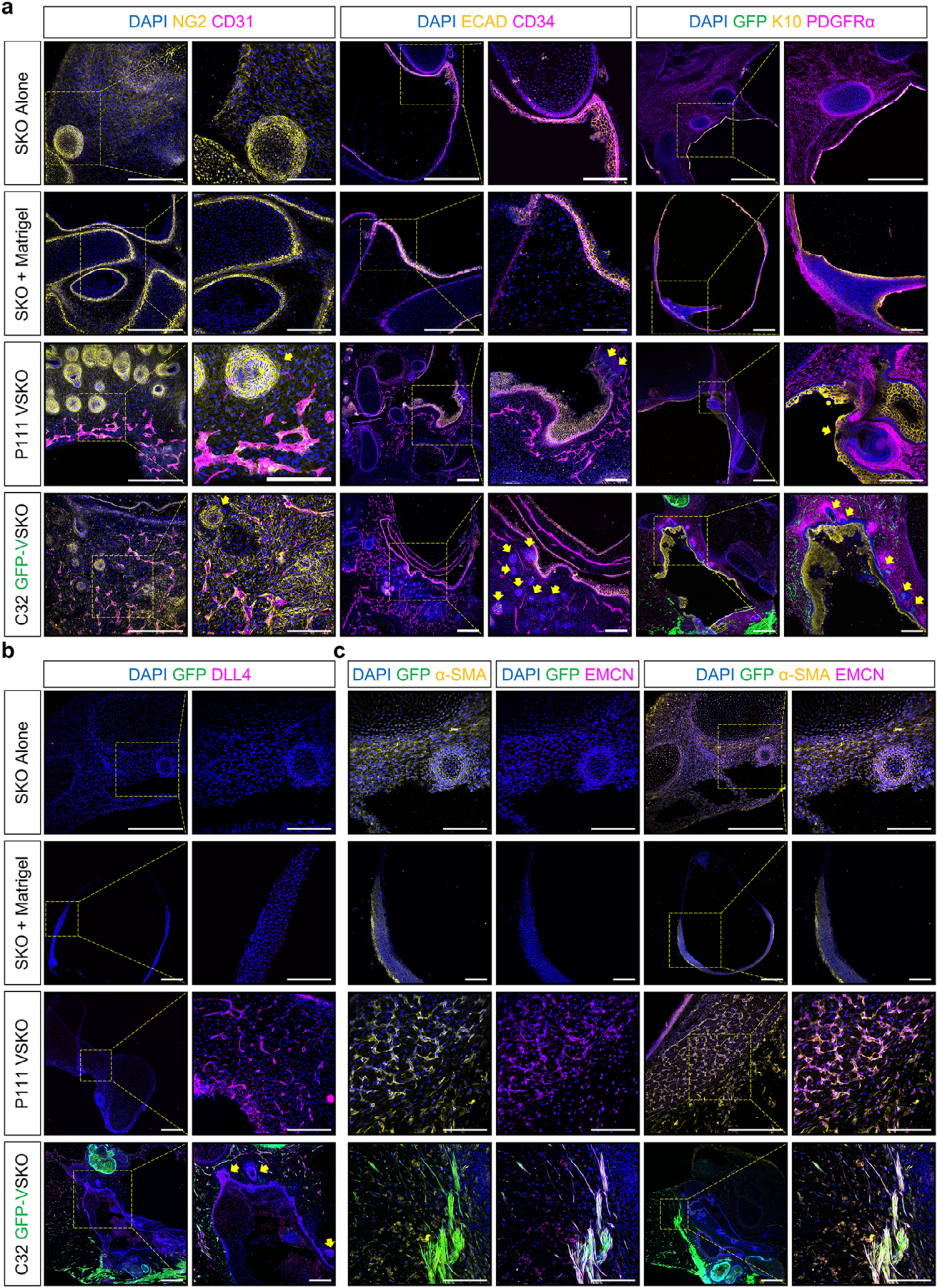
Characterization of Vascularized Human Skin Organoids. a) Representative immunofluorescence microscopy images of day 115 P111 and C32 human induced pluripotent stem cell (hiPSC)‐derived vascularized skin organoids (VSKO) with control groups. Neural/glial antigen 2 (NG2, yellow color) antibody characterizes pericyte capillary formation when stained with cluster of differentiation (CD)31 (magenta color), which shows endothelial capillary formation, as well as CD34 (magenta color). Terminally differentiated keratinocytes are cytokeratin 10^+^ (K10, yellow color), epithelium is epithelial cadherin^+^ (ECAD, yellow color), dermal region is platelet‐derived growth factor receptor α^+^ (PDGFRα, magenta color). The P111 VSKO contains two green fluorescent protein (GFP)^−^ vascular organoids (VO) co‐cultured with one GFP^−^ skin organoid (SKO), while the C32 VSKO group includes two GFP‐tagged VOs co‐cultured with one GFP^−^ SKO. Scale bars are 500 µm. Magnified image scale bars are 200 µm. b) Representative immunofluorescence microscopy images of day 115 P111 and C32 hiPSC‐derived VSKOs with control groups. The P111 VSKO contains two GFP^−^ VOs co‐cultured with one GFP^−^ SKO, while the C32 VSKO group includes two GFP‐tagged VOs co‐cultured with one GFP^−^ SKO. All delta‐like protein 4^+^ (DLL4, magenta color) capillaries are also GFP^+^, showing arterial differentiation from the VOs. c) Representative immunofluorescence microscopy images of day 115 P111 and C32 hiPSC‐derived VSKOs with control groups. All endomucin^+^ (EMCN, magenta color) capillaries are also GFP^+^, highlighting venous differentiation from the VOs. All α‐smooth muscle actin^+^ (α‐SMA, yellow color) capillaries are also GFP^+^, demonstrating the differentiation of vascular smooth muscle cell‐derived capillaries from the VOs. Scale bars are 500 µm. Magnified image scale bars are 200 µm. Yellow colored arrows indicate hair follicles. Yellow color boxes indicate regions of interest. Cell nuclei are stained with 4′,6‐diamidino‐2‐phenylindole (DAPI, blue color).

VSKOs demonstrated CD34^+^ and CD31^+^/NG2^+^ capillaries. (**Figure** **6**a; Video S2, Supplementary Video 2). These capillaries showed a nice distinction, separate from the ECAD^+^ epithelium, recapitulating vascularized skin as seen in vivo (Figure 6a).

Further characterization demonstrated that the endothelial capillaries were structurally supported by NG2^+^ pericytes and contained α‐SMA^+^ mural cells (Figure 6a,c). Additionally, endothelial differentiation occurred, where DLL4^+^ arterial capillaries and EMCN^+^ venous capillaries were observed (Figure 6b,c). Comparatively, both the SKO + Matrigel and SKO Alone control groups did not show any CD34^+^ or CD31^+^/NG2^+^ capillary‐like structures for both cell lines (Figure 6a–c). This was validated with the GFP^+^ VOs co‐cultured with a GFP^−^ SKO in the C32 cell line (Figure 6a–c). Consequently, this indicated that the SKOs were vascularized by the VOs in the co‐culture setting.

### 2.7 Vascularized Human Skin Organoids Demonstrate Perifollicular Vessel Formation

**Figure 7:**
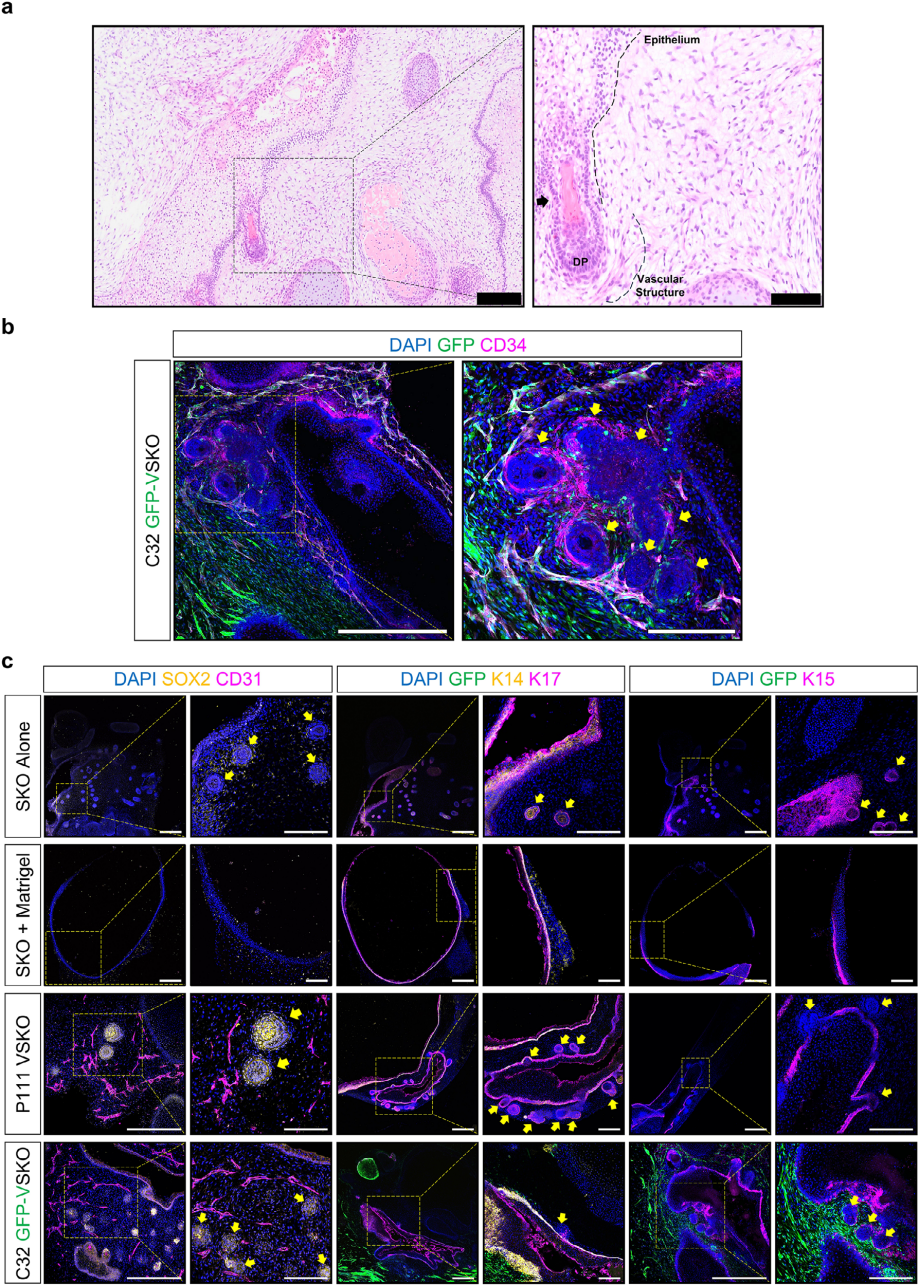
Hair Follicle Characterization of Vascularized Human Skin Organoids. a) Representative hematoxylin & eosin staining images of paraffin‐embedded sections of day 115 P111 human induced pluripotent stem cell (hiPSC)‐derived vascularized skin organoid (VSKO). Scale bar is 200 µm. Magnified image scale bar is 100 µm. Black color box indicates the region of interest. Black color arrow highlights a hair follicle. b) Representative immunofluorescence microscopy images of day 115 C32 hiPSC‐derived VSKOs. Two green fluorescent protein (GFP)‐tagged vascular organoids (VO) are co‐cultured with one GFP^−^ skin organoid (SKO) and stained for cluster of differentiation (CD)34 (magenta color), demonstrating perifollicular vascularization. Scale bar is 500 µm. Magnified image scale bar is 200 µm. c) Representative immunofluorescence microscopy images of day 115 P111 and C32 hiPSC‐derived VSKOs with control groups. The P111 VSKO contains two GFP^−^ VOs co‐cultured with one GFP^−^ SKO, while the C32 VSKO group includes two GFP‐tagged VOs co‐cultured with one GFP^−^ SKO. SKOs and VSKOs were stained with the following antibodies: SRY‐box transcription factor 2 (SOX2, yellow color), CD31 (magenta color), cytokeratin 14 (K14, yellow color), cytokeratin 15 (K15, magenta color), and cytokeratin 17 (K17, magenta color). Scale bars are 500 µm. Magnified image scale bars are 200 µm. Cell nuclei are stained with 4′,6‐diamidino‐2‐phenylindole (DAPI, blue color). Yellow color boxes indicate regions of interest. Yellow colored arrows indicate hair follicles. Dermal papillae (DP).

Compared to the endothelialized SKO model (Figure 2c), hair follicles and keratinization were observed in VSKOs derived from both hiPSC lines (**Figure** **7**a–c). Furthermore, hematoxylin and eosin staining demonstrated the formation of a thick epithelium, hair follicles, and dermal papillae within the dermis, consistent with previous reports on SKOs (Figure 7a).^[^ ^32^ ^]^ Immunofluorescence microscopy analyses confirmed that endothelial capillaries had penetrated the perifollicular regions of hair follicles (Figure 7a,b; Video S3, Supplementary Video 3). There is a heterogeneous population of CD34⁺ and CD34^−^ cells within VSKOs, with vascular‐like structures exclusively formed by GFP⁺/CD34⁺ cells, indicating the contribution of VOs to vascularization (Figure 7b). In the absence of VOs (Figure 7c, SKO Alone and SKO + Matrigel control groups), no vascular structures or CD31⁺ cells were detected, indicating the critical role VOs demonstrate in vascularizing VSKOs.

In contrast to endothelialized skin organoids using ECFCs, both P111 and C32 hiPSC‐derived VSKOs developed thick skin layers containing hair follicles, suggesting the clear integration of vascularization and their supportive effect on skin layer formation. Figure 7c also shows that the hair follicles in VSKOs are SRY‐box transcription factor 2 (SOX2)^+^, K14^+^, cytokeratin 15 (K15)^+^, and K17^+^, as seen in the current SKO model.^[^ ^25^, ^26^ ^]^ However, the SKO + Matrigel group did not contain any hair follicle structures (Figure 7c).

### 2.8 Vascularized Human Skin Organoids Contain Immune Cells

**Figure 8:**
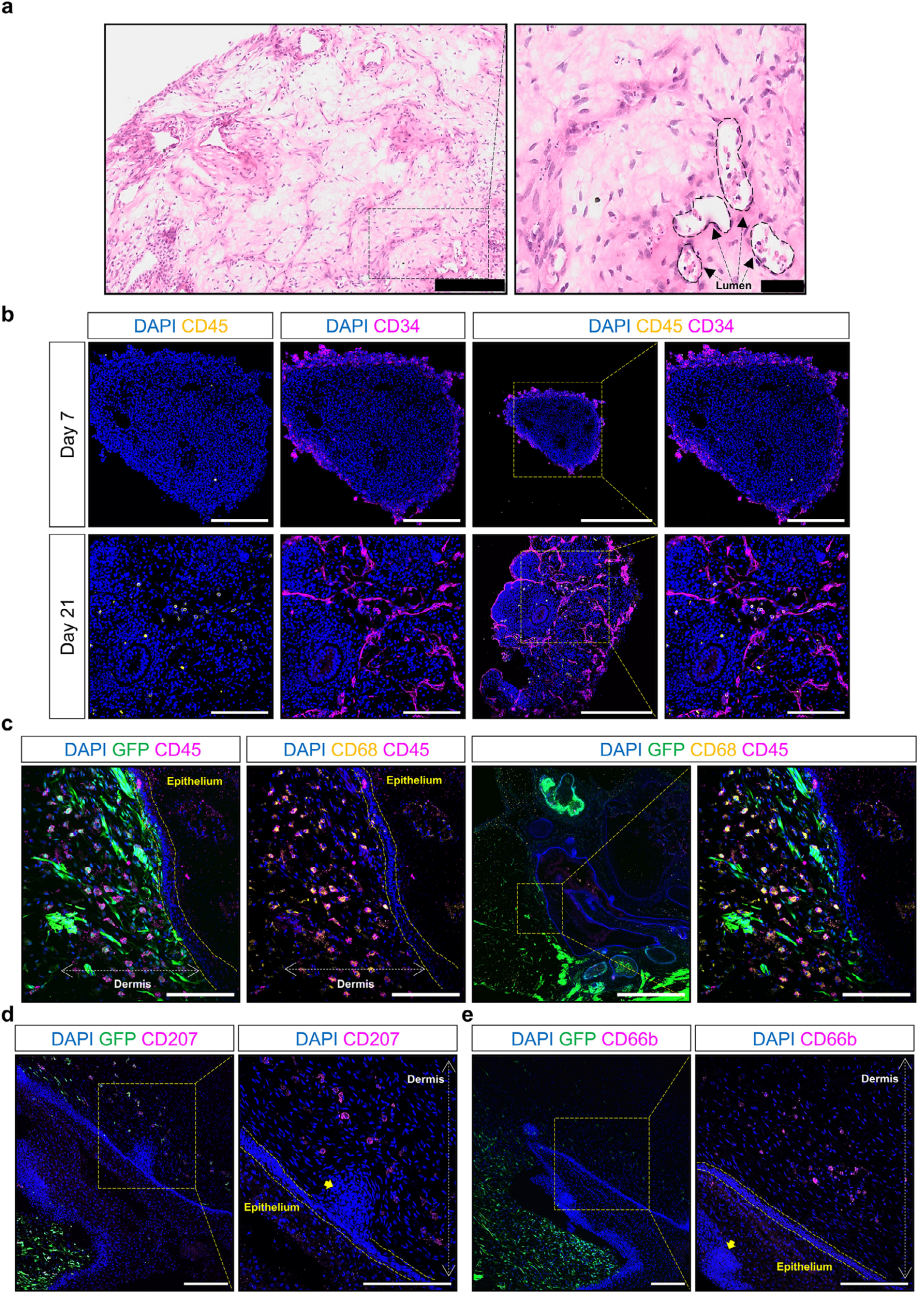
Vascular Organoid‐Derived Immune Cells Migrate into Skin Organoids. a) Representative hematoxylin & eosin staining images of paraffin‐embedded sections of day 40 P111 human induced pluripotent stem cell (hiPSC)‐derived vascular organoid (VO) shows the development of vascular‐like structures containing immune‐like cells. Scale bar is 200 µm. Magnified image scale bar is 50 µm. Black color box indicates the region of interest. b) Representative immunofluorescence microscopy images of day 7 and day 21 P111 hiPSC‐derived VOs. While cluster of differentiation (CD)45^+^ cells (yellow color) are not present at day 7, their numbers increase, and they disperse throughout the VOs by day 21. Notably, some CD45^+^ cells are also positive for CD34 (magenta color). Scale bar is 200 µm. c) Representative immunofluorescence microscopy images of day 115 C32 hiPSC‐derived vascularized skin organoid (VSKO). The VSKO contains two green fluorescent protein (GFP)‐tagged VOs co‐cultured with one GFP^−^ SKO. These images highlight the presence of CD45^+^ (magenta color) and CD68^+^ (yellow color) cells within the dermal layer of the VSKO. Scale bar is 1 mm. Magnified image scale bar is 200 µm. d) Representative immunofluorescence microscopy images of day 115 C32 hiPSC‐derived VSKO show the presence of CD207^+^ (magenta color) cells within the dermal layer next to a hair follicle. Scale bars are 200 µm. e) Representative immunofluorescence microscopy images of day 115 C32 hiPSC‐derived VSKO demonstrate the presence of CD66b^+^ (magenta color) cells within the dermal layer. Scale bars are 200 µm. Cell nuclei are stained with 4′,6‐diamidino‐2‐phenylindole (DAPI, blue color). Yellow color boxes indicate regions of interest. Yellow color arrows indicate hair follicles.

Previous reports described the absence of immune and vascular components during hiPSC‐derived SKO differentiation.^[^ ^25^, ^26^, ^33^ ^]^ Here, we evaluated the presence of immune cells within VSKOs. VOs developed vascular‐like structures that contained immune cell‐like phenotypes within their luminal structures (**Figure** **8**a). Therefore, we explored the presence of immune cells within VOs and VSKOs. Immunofluorescence microscopy confirmed the presence of a hematopoietic cell lineage in VOs by day 21 of differentiation, where we observed CD34^+^/CD45^+^ positive cells next to the CD34^+^ vascular structures (Figure 8b). Although CD45^+^ cells were not visibly present in VOs at day 7, their numbers significantly increased in the subsequent weeks and dispersed throughout the organoids after the visible generation of hematopoietic cells on day 21 of VO differentiation (Figure 8b).

We therefore explored the transfer of hematopoietic cells from the VO to the VSKO. The infiltration of CD45^+^ hematopoietic cells to the skin components of VSKOs was confirmed with immunofluorescence microscopy (Figure 8c). It was observed that the CD45^+^ cells were homogenously distributed throughout the entire VSKO, predominantly in the dermis with a few cells only in the epidermis. All CD45^+^ cells were GFP^+^ as we co‐cultured two GFP^+^ VOs with one GFP^−^ SKO (Figure 8c). Consequently, the VSKO co‐culture demonstrated the formation and migration of CD45^+^ immune cells from the VO into the SKO.

Macrophages and Langerhans cells are key components of skin tissue remodeling and immune regulation; therefore, we assessed their presence in VSKOs by performing immunofluorescence microscopy. The presence of CD68^+^ cells in the dermal region of VSKOs was confirmed, where almost all CD45^+^ cells were CD68^+^ (Figure 8c). Furthermore, other immune cells at this differentiation timepoint of the VSKO were identified as CD207^+^, demonstrating the presence of either migrating Langerhans cells from the dermis to the epidermis or dermal dendritic cells that were surrounding nearby hair follicles. We also observed CD66b^+^ neutrophils in the VSKO dermis (Figure 8d,e).

## 3 Discussion

Autologous skin graft transplantation is an effective approach for treating major cutaneous wounds.^[^ ^55^ ^]^ Although autologous skin grafts are effective in supporting wound closure and reducing contracture, their therapeutic benefits are limited by their inconsistency in graft take, lack of donor sites, and high metabolic demands requiring considerable vascular integration.^[^ ^55^ ^]^ Additionally, cultured epithelial autografts demonstrate poor infection tolerance due to inferior vascular perfusion, as they only close the epidermal barrier.^[^ ^56^, ^57^ ^]^ Physiologically, vasculature enables the diffusion of nutrients and metabolic exchange to the superficial layers of skin.^[^ ^11^ ^]^ This highlights the importance of developing vascularized skin substitutes as they mitigate infection and necrosis.^[^ ^41^, ^58^ ^]^

A key aspect of skin function is its vasculature, which enables metabolic exchange and plays a key role in immune function. Previous human SKO models were devoid of vascularization and immune cells.^[^ ^25^, ^26^, ^28^, ^29^, ^30^, ^31^, ^32^, ^33^, ^34^, ^35^, ^36^, ^37^, ^38^, ^59^ ^]^ During human development, skin is derived from the surface ectoderm, while blood vessels and the immune cells are both derived from the mesoderm.^[^ ^51^, ^60^, ^61^ ^]^ In the current study, we have developed a VSKO from hiPSCs and showed its superiority to our current SKO and endothelialized SKO models.

The conceptualization of adding VEGF to OMM enabled the prolific generation and maintenance of VOs, VSKOs, and sustained VO‐derived immune cell differentiation. Considering blood vessels promote transplant survival, the impact of vascularization on organoids survival after transplantation should be further investigated. Here, we established VSKOs from two different hiPSC lines derived from different origins, skin fibroblasts (C32 line) and CD34^+^ human fetal placental cells (P111 line) and demonstrated the reproducibility of the differentiation protocol among different hiPSC lines.

We began the co‐culture with GFP‐tagged ECFCs on day 18 of SKO differentiation to recapitulate vasculogenesis of a developing human embryo.^[^ ^62^, ^63^ ^]^ The vascularization process was modulated by the addition of VEGF. Considering the importance of pericytes and VSMCs in vasculature, endothelial cells alone are insufficient in recapitulating blood vessel structures.^[^ ^64^, ^65^ ^]^ Interestingly, the NG2^+^ mesenchymal cells of the SKO migrated toward the ECFCs to structurally support them after co‐culture. This supports the fact that ECFCs express key chemokines that stimulate mesenchymal stem cell migration via paracrine signaling.^[^ ^66^ ^]^ Further characterization is required to understand the molecular mechanisms behind ECFC‐SKO cellular interactions. Endothelial cells have been used to endothelialize different organoids.^[^ ^67^, ^68^ ^]^ However, there are identified limitations in the application of endothelial cells, including poor integration, and limited vascular stability which hinder the formation of functional vasculature *in vitr* *o*. In addition, the use of ECFCs with SKOs introduces an allogenic component, as they are harvested from unrelated human placentas. This challenges downstream applications such as grafting, as this increases the chance for immune rejection.

Furthermore, the GFR‐M droplet prevented hair follicle development in the SKO + GFP‐ECFC and SKO + Matrigel groups, when compared to the SKO Alone control group. Also, there was an absence of keratinization in the SKO + GFP‐ECFC group. In addition, we observed increased levels of cartilage and mesenchymal lineage formation in SKOs receiving ECFCs that may be attributed to the secretion of growth factors such as platelet‐derived growth factor and transforming growth factor‐β by ECFCs, that promote mesenchymal lineage differentiation.^[^ ^69^, ^70^, ^71^ ^]^ Overall, these limitations were significant enough to investigate other methodologies of vascularizing SKOs, that can maintain effective hair follicle development and keratinization.

Similarly to the endothelialized SKO, we initiated a co‐culture of two VOs with one SKO on day 18 of SKO differentiation with the addition of VEGF to recapitulate vasculogenesis of a human embryo.^[^ ^63^ ^]^ Remarkably, the vascular‐like structures of the VSKOs were quite robust and diverse, demonstrating lengths significantly greater than 1 mm, with structural support from NG2^+^ pericytes, and showed α‐SMA^+^ VSMCs. Our investigation showed that the mural cells are efficiently provided by the VOs. These blood vessel‐like structures also contained a lumen housing immune cells, similarly, as seen in vivo.^[^ ^72^ ^]^ Previous studies have shown that the vasculogenic potential of endothelial progenitors is enhanced when combined with a mesenchymal component.^[^ ^64^, ^73^ ^]^ This may explain the improved vascularization and maturation observed in VSKOs.

The presence of hair follicles in VSKOs, but not in endothelialized SKOs, is likely attributed to differences in the vascularization strategy and co‐culture conditions. The SKO + GFP‐ECFC co‐culture was conducted in a floating culture system, which is suboptimal for skin folliculogenesis. The VSKO experiments were maintained in an ALI co‐culture system, which is known to promote appendage formation. While both systems used a GFR‐M droplet, the ALI conditions in VSKO may minimize their negative effects. In addition, VOs not only provide endothelial cells, like the SKO + GFP‐ECFC group, but also perivascular and stromal components that better resemble the dermal niche and may provide essential inductive signals for hair follicle development. Consequently, this model enhances the potential applications of vascularized skin constructs for regenerative medicine applications and opens new avenues for the complete in vitro modelling of human skin.

To further improve the vascularized SKO model, we generated VOs from the same hiPSC cell line as SKOs. Our results showed the development of endothelial, mural, hematopoietic, and mesenchymal cell populations within the VO. Considering that VOs demonstrate a heterogenous population of different cell types, we can investigate different methods of vascularization and understand the role of each cell involved in this process. VOs enable the evaluation of transplantation regimens, and investigation of immune‐mediated diseases, inflammation, and tissue repair. Utilizing VOs as a method to generate VSKOs creates a robust platform for understanding associated skin diseases, transplantation methodologies and drug screening opportunities. Comparatively to current SKO models that are devoid of immune cells and vasculature, VSKOs provide access to evaluating the fidelity of vasculature and inflammatory diseases, as well as new immune cell and vasculature targeting drugs in pre‐clinical development.

Additionally, endothelial differentiation was evaluated with an observation of DLL4^+^ arterial capillaries and EMCN^+^ venous capillaries, highlighting the appropriate recapitulation of a blood vessel as seen in vivo.^[^ ^74^, ^75^ ^]^

Whilst the VSKOs were co‐cultured on an ALI to recapitulate the external environment human skin is exposed to, SKOs appeared to maintain hair follicle development after vascularization, where perifollicular vascularization was consistently observed throughout the VSKO as seen in vivo.^[^ ^75^ ^]^ The ALI culture, facilitated using Transwell system, has previously shown to enhance hair follicle development in SKOs.^[^ ^33^ ^]^ The recapitulation of hair follicles in our VSKO model can further the potential of current in vitro models. VSKOs can also be used as a tool to investigate the interaction of hair follicles with vasculature, as well as hair‐related pathologies.^[^ ^75^, ^76^ ^]^ While hair follicle development did not appear to be impaired upon co‐culture; further studies should be conducted to assess the impact of vascularization on folliculogenesis levels.

The presence of a hematopoietic lineage within VSKOs allows the study of inflammatory skin disorders (e.g., psoriasis and atopic dermatitis), autoimmune conditions (e.g., scleroderma), and skin genetic disorders with unmet clinical needs (e.g., hidradenitis suppurativa, Netherton syndrome, and epidermolysis bullosa). While future development is needed to fully replicate the complexity of skin immunology, the current immune cell populations within the VSKOs mark a significant step toward establishing physiologically relevant in vitro skin platforms.

Considering this study is the first of its kind, we were able to observe the migration of CD45^+^ immune cells from VO into VSKO, where they exhibited the morphology of CD68^+^ macrophages, CD207^+^ immune cells, and CD66b^+^ neutrophils. This observation is consistent with the fact that macrophage recruitment to the skin begins during primitive hematopoiesis in the yolk sac, where they mature into skin‐resident cells.^[^ ^77^ ^]^ Langerhans cells are a unique cell population to the skin organ, where fate‐mapping of macrophages demonstrates in situ proliferation to Langerhans cells in the skin during embryonic development.^[^ ^78^, ^79^, ^80^, ^81^ ^]^ Mechanistically, this is driven by interleukin‐34 release via macrophage‐colony stimulating factor receptor.^[^ ^78^ ^]^

The observation of CD207^+^ immune cells is consistent with hematopoiesis and macrophage differentiation within the VSKO. Notably, this observation aligns with previous studies that mature human SKOs resemble human fetal craniofacial skin at 18‐week of gestation.^[^ ^26^, ^78^ ^]^ On day 115 of differentiation (≈16‐week of embryonic development), the presence of CD207^+^ immune cells in the dermal region suggests two possibilities: first, the migration of Langerhans cell precursors from the dermis^[^ ^82^, ^83^ ^]^; and second, the presence of dermal dendritic cells,^[^ ^82^, ^83^ ^]^ both of which express CD207^+^. Thus, further studies at extended time points of VSKO differentiation are required to determine the specific identity and function of these immune cells. Considering the immune cells are predominantly myeloid‐derived, the observation of CD66b^+^ cells is consistent with VO differentiation of unique immune cell types that support vascular function.^[^ ^8^, ^84^, ^85^ ^]^ Moving forward, this will require further investigation to claim their sub‐population phenotypes.

While drug screening studies were not conducted in this study, the vascularized skin models developed here represent a versatile platform for “clinical trials in a dish” studies. VSKOs offer future utility in therapies targeting skin vascularization, wound healing, and other dermatological disorders. In addition, this VSKO model demonstrates potential for immunotherapy drug development and disease modelling, considering drug screening is becoming increasingly popular for new therapeutic strategies, and proves to be a potential future application of the VSKO model.

## 4 Conclusion

Currently, SKOs lack vasculature and immune cells in a physiologically relevant context.^[^ ^25^, ^35^, ^36^, ^37^, ^38^ ^]^ To address this limitation, we developed a VSKO from hiPSCs and showed its superiority to our endothelialized SKO model. Generating and characterizing VSKOs on an ALI culture system recapitulated vascularized human skin in a physiologically relevant environment, which demonstrated macrophages, CD207^+^ immune cells, neutrophils, and perifollicular vasculature with appropriate luminal structures. These VSKOs can be used for a range of experimental purposes such as regenerative medicine, drug screening, disease modelling, developmental biology, and biomarker discovery.

## 5 Experimental Section

### Generation and Maintenance of Human Induced Pluripotent Stem Cell Lines

In this study, two hiPSC lines referred to as P111 and C32, were used to generate SKOs, VOs, and VSKOs. The P111 and C32 cell lines were generated and maintained as previously described (HREC/09/QRBW/14).^[^ ^25^, ^32^, ^86^ ^]^

### Skin Organoid Differentiation

hiPSC‐SKOs were generated and maintained in OMM as previously described.^[^ ^25^, ^32^ ^]^

### Endothelialized Skin Organoid Co‐Culture

Day 12 of SKO Differentiation: ECFCs derived from CD34^+^ human fetal placental cells (HREC/09/QRBW/14), which were transfected with GFP, were cultured with EGM2, Lonza, CC‐3162 containing 1% penicillin‐streptomycin (Gibco, 15140122), that was coated with a 1:100 dilution of Collagen, Type 1 solution from rat tail (Sigma‐Aldrich, C3867) in UltraPure DNase/RNase‐Free Distilled Water (Invitrogen, 10977015), and was incubated for 2 h at room temperature, to allow the coat to set.

Day 13–18 of SKO Differentiation: The ECFC culture medium gradually changed. A daily 20% reduction of EGM2, and daily 20% increase of OMM with 5 ng/mL VEGF, PeproTech, 100–20 was performed, to help ECFCs adjust to the SKO co‐culture medium.

Day 18 of SKO Differentiation: 150,000 ECFCs were homogenized in 15 µL of GFR‐M, Corning, 354230, and pipetted over the SKOs. Afterward, the co‐cultured SKOs were incubated at 37 °C (5% CO_2_) for 15 min to polymerize the GFR‐M. The SKO + GFP‐ECFC co‐culture was transferred to a 24‐well clear flat bottom ultra‐low attachment multiple well plate (Costar, 3473), containing 500 µL OMM with 50 ng mL^−1^ VEGF. The control group was one SKO covered with a 15 µL GFR‐M droplet with 500 µL OMM with 50 ng mL^−1^ VEGF (SKO + Matrigel). A reference control group of the current SKO model was included, which was one SKO cultured with 500 µL OMM only (SKO Alone). All groups were incubated at 37 °C (5% CO_2_). Half medium changes were performed every 3 days (from day 18–30) and every 2 days from day 30 until day 115. Organoids were imaged on an Olympus IX73 Inverted Microscope, with Olympus cellSens Imaging software, version 1.8. Images were prepared with Fiji software, version 1.54f.

### Vascular Organoid Differentiation

The differentiation of VOs was adapted from previous studies.^[^ ^51^, ^54^ ^]^

Day ‐2 of VO Differentiation: 9,000 live hiPSCs were transferred to a 96‐well clear round bottom ultra‐low attachment microplate (Corning, 7007), with 150 µL of mTeSR Plus medium (STEMCELL Technologies, 05825) containing 10 µM StemMACS Y27632 (ROCKi, rho‐associated kinase (ROCK) inhibitor, Miltenyi Biotec, 130‐106‐538), and 1% antibiotic‐antimycotic (Gibco, 15240096). The plates were then centrifuged to form 3D aggregates and were incubated at 37 °C (5% CO_2_).

Day 0 of VO Differentiation: All medium was removed, and 150 µL of STEMdiff APEL2 medium (APEL2, STEMCELL Technologies, 05270) containing: 8 µM Chir99021 (BioGems, 2520691), 25 ng mL^−1^ BMP‐4 (PeproTech, AF‐120‐05ET), and 1% antibiotic‐antimycotic was added and incubated at 37 °C with 5% CO_2_.

Day 2 of VO Differentiation: All medium was removed and 150 µL of APEL2 medium containing: 10 µM SB431542 (STEMCELL Technologies, 72234), 10 ng mL^−1^ fibroblast growth factor 2 (FGF‐2, Miltenyi Biotec, 130‐093‐841), 100 ng mL^−1^ VEGF, and 1% antibiotic‐antimycotic was added.

Day 5 of VO Differentiation: All medium was removed and 150 µL of EGM2‐Microvascular medium (CC‐3202) containing 10 µM SB431542, 100 ng mL^−1^ VEGF, and 1% antibiotic‐antimycotic was added.

Day 7 of VO Differentiation: A 15 µL GFR‐M droplet was pipetted onto the VOs and then incubated at 37 °C (5% CO_2_) for 15 min, to polymerize the GFR‐M. Afterward, VOs were transferred to a 24‐well clear flat bottom ultra‐low attachment multiple well plate with EGM2‐Microvascular medium containing: 50 ng mL^−1^ VEGF, 100 ng mL^−1^ FGF‐2, and 1% antibiotic‐antimycotic. Half medium changes were performed every other day.

Day 15 of VO Differentiation: A full medium change was performed, where 500 µL of OMM with 50 ng mL^−1^ VEGF was added to each well. Half medium changes were performed every 3 days (from day 18–30) and every 2 days from day 30, until day 115, where the final concentration of VEGF was reduced from 50 to 20 ng mL^−1^. Organoids were imaged on an Olympus IX73 Inverted Microscope, with Olympus cellSens Imaging software, version 1.8. Images were prepared with Fiji software, version 1.54f.

### Vascularized Skin Organoid Co‐Culture

Day 1 of SKO Differentiation: hiPSCs were aggregated for VO development (Day ‐2 of VO Differentiation).

Day 10 of SKO Differentiation: A 5 µL GFR‐M droplet was pipetted onto the VOs and incubated at 37 °C (5% CO_2_) for 15 min to polymerize the GFR‐M. Afterward, VOs were transferred to a 24‐well clear flat bottom ultra‐low attachment multiple well plate with EGM2‐Microvascular medium containing: 50 ng mL^−1^ VEGF, 100 ng mL^−1^ FGF‐2, and 1% antibiotic‐antimycotic.

Day 18 of SKO differentiation: One SKO was placed on a Transwell insert (Corning, 3450). The Transwell insert was placed on top of 1.5 mL OMM with 50 ng mL^−1^ VEGF. Two VOs were placed adjacent to the SKO, where a 30 µL GFR‐M droplet was pipetted onto the three organoids and then incubated at 37 °C (5% CO_2_). This group is considered the VSKO group. The control group was one SKO in a 10 µL GFR‐M droplet, containing OMM with 50 ng mL^−1^ VEGF (SKO + Matrigel). A reference control group of the current SKO model was included, which was one SKO with OMM only (SKO Alone). Half medium changes were performed every 3 days (from day 18–30) and every 2 days from day 30 until day 115, where the final concentration of VEGF for the VSKO and SKO + Matrigel group was reduced to 20 ng mL^−1^. Organoids were imaged on an Olympus IX73 Inverted Microscope, with Olympus cellSens Imaging software, version 1.8. Images were prepared with Fiji software, version 1.54f.

### Organoid Fixation and Histology

Organoid samples were harvested and prepared for immunofluorescence microscopy as previously described.^[^ ^25^ ^]^ Cryosections were cut at a thickness of 25 µm.

### Cryosection Immunofluorescence Microscopy

Fixed organoid cryosections were stained and imaged as previously described with key antibody markers using 10% normal goat serum (Invitrogen, 31873), or 10% normal donkey serum (Sigma‐Aldrich, D9663) (Tables S1 and S2, Supporting Information).^[^ ^25^ ^]^

### Hematoxylin and Eosin Staining

Organoid samples were prepared and imaged as previously described.^[^ ^25^ ^]^ Paraffin‐embedded sections were cut at a thickness of 4 µm.

### Whole Mount Immunofluorescence Microscopy

The organoids were washed with phosphate‐buffered saline (PBS, Gibco, 18912‐014), and then permeabilized with blocking buffer containing: PBS, 10% normal goat serum, and 0.3% Triton X‐100 (Sigma‐Aldrich, X‐100). Samples were incubated for 2 h on a shaker at room temperature and then moved to 4 °C overnight on a shaker. Afterward, an antibody binding buffer solution containing: PBS, 10% normal goat serum, 0.1% Triton X‐100, and key antibody markers (Tables S1 and S2, Supporting Information) were added and incubated at 4 °C for 2 days on a shaker, away from light. After, samples were washed with washing buffer solution: PBS with 0.1% Triton X‐100. Secondary antibodies were added to the samples for 2 h on a shaker at room temperature and then moved to 4 °C overnight on a shaker, away from light. Samples were washed again with the washing buffer, and a 1:1000 dilution of 4′,6‐diamidino‐2‐phenylindole (DAPI, Invitrogen, D1306) in PBS was added to the samples and incubated for 3 h at room temperature on a shaker, away from light. Afterward, the samples were washed with PBS, and RapiClear (SUNJin Lab, RC152001) was added onto the organoids, on a 35 mm dish (MatTek Life Sciences, P35G‐1.5‐14‐C) and incubated overnight at room temperature.

Samples were imaged with a Nikon/Spectral Spinning Disc Confocal Microscope with NIS‐Elements AR Imaging Software, version 5.11.03. Videos were prepared with Imaris software (Oxford Instruments), version 9.9.1.

### Flow Cytometric Analysis of Vascular Organoids

VOs were washed with PBS and mechanically chopped with a scalpel, whilst using 100% Accutase solution (Sigma‐Aldrich, A6964) to assist with digestion. The organoid pieces were incubated at 37 °C on a shaker for 30 min. After centrifugation, the digested organoids were strained through a 70 µm cell strainer (Falcon, 352350). After counting, the cells were blocked with a 1:5 dilution of Fc receptor blocking reagent, human (Sigma‐Aldrich, 130‐059‐901), and stained with key antibody markers (Table S3, Supporting Information), diluted in fluorescence‐activated cell sorting (FACS) solution containing: PBS, 0.5% bovine serum albumin (Sigma‐Aldrich, A9418), and 0.5 m ethylenediamine tetra acetic acid (Sigma‐Aldrich, 20–518) for 30 min at 4 °C. Samples were washed with FACS solution and excess/unbound antibodies were removed after centrifugation. After, samples were stained with a 1:100 dilution of 7‐AAD viability staining solution (BioLegend, 420404). Samples were analyzed on a BD LSRFortessa X‐20 Cell Analyzer (BD Biosciences) and prepared with FlowJo software, version 10.8.1.

## Conflict of Interest

The authors declare no conflict of interest.

## Supporting information



Supporting Information

Supplementary Video1

Supplementary Video2

Supplementary Video3

## Data Availability

The data that support the findings of this study are available from the corresponding author upon reasonable request.
