# Supplementary material for: Coordinated Development of Immune Cell Populations in Vascularized Skin Organoids from Human Induced Pluripotent Stem Cells
Source: Adv Healthc Mater. 2025 Aug 16;14(31):e02108. doi: 10.1002/adhm.202502108 (PMC12683213; doi:10.1002/adhm.202502108)
Supplement: Supplementary file 1 — Supporting Information [file ADHM-14-0-s001.docx]

**Supplementary Materials**

***Supplementary Table S1***: List of Primary Antibodies Used for Immunofluorescent Staining.

| **Primary Antibody Name** | **Antibody Target** | **Host Species** | **Company & Catalogue Number** | **Dilution** |
| --- | --- | --- | --- | --- |
| Anti-Alpha Smooth Muscle Actin Antibody | Monoclonal to Human Alpha Smooth Muscle Actin (α-SMA) | Mouse | Abcam, ab7817 | 1:100 |
| Anti-Alpha Smooth Muscle Actin Antibody | Polyclonal to Human Alpha Smooth Muscle Actin (α-SMA) | Rabbit | Abcam, ab5694 | 1:100 |
| Anti-Cleaved Caspase-3 | Polyclonal to Human Cleaved Caspase-3 (CC3) | Rabbit | Cell Signaling Technology, 9661 | 1:100 |
| Anti-CD31 Antibody | Monoclonal to Human CD31 (CD31) | Mouse | Abcam, ab24590 | 1:100 |
| Anti-CD34 Antibody | Monoclonal to Human CD34 (CD34) | Rabbit | Abcam, ab81289 | 1:100 |
| Anti-CD45 Antibody | Monoclonal to Human Receptor-Type Tyrosine-Protein Phosphatase C (CD45) | Mouse | Abcam, ab33533 | 1:100 |
| Purified Anti-CD66b Antibody | Monoclonal to Human CD66b (CD66b) | Mouse | BioLegend®, 305102 | 1:100 |
| Anti-CD68 Antibody | Monoclonal to Human CD68 (CD68) | Mouse | Invitrogen^TM^,  14-0688-82 | 1:50 |
| Anti-CD68 Antibody | Monoclonal to Human CD68 (CD68) | Rabbit | Cell Signaling Technology, 76437 | 1:100 |
| PE Anti-CD207 Antibody | Monoclonal to Human/Mouse CD207 (CD207) | Mouse | BioLegend®, 144203 | 1:100 |
| Anti-DLL4 Antibody | Monoclonal to Mouse Delta-Like Protein 4 (DLL4) – Cross-Reacts with Human DLL4 | Goat | R&D Systems, AF1389 | 1:50 |
| Anti-E-Cadherin Antibody | Monoclonal to Human Epithelial-Cadherin (ECAD) | Mouse | BD Transduction Laboratories, 610181 | 1:100 |
| Anti-Endomucin Antibody | Polyclonal to Human Endomucin (EMCN) | Rabbit | Invitrogen^TM^, PA5-115178 | 1:100 |
| Anti-Cytokeratin 10 Antibody | Monoclonal to Human Cytokeratin 10 Cytoskeleton Marker (K10) | Mouse | Abcam, ab9026 | 1:100 |
| Anti-Cytokeratin 14 Antibody | Monoclonal to Human Cytokeratin 14 (K14) | Mouse | Abcam, ab7800 | 1:50 |
| Anti-Cytokeratin 14 Antibody | Monoclonal to Human Cytokeratin 14 Cytoskeletal Marker (K14) | Rabbit | Abcam, ab181595 | 1:100 |
| Anti-Cytokeratin 15 Antibody | Monoclonal to Human Cytokeratin 15 (K15) | Mouse | GeneTex, GTX72325 | 1:50 |
| Anti-Cytokeratin 17 Antibody | Monoclonal to Human Cytokeratin 17 Cytoskeletal Marker (K17) | Rabbit | Abcam, ab109725 | 1:100 |
| Anti-NG2 Antibody | Monoclonal to Human Neuron/Glial Antigen 2 (NG2) | Rabbit | Abcam, ab275024 | 1:100 |
| Anti-PDGF Receptor Alpha Antibody | Polyclonal to Human CD140a (PDGFRα) | Rabbit | Abcam, ab61219 | 1:50 |
| Anti-SOX2 Antibody | Polyclonal to Human SRY-Box Transcription Factor 2 (SOX2) | Rabbit | Abcam, ab97959 | 1:100 |

***Supplementary Table S2***: List of Secondary Antibodies Used for Immunofluorescent Staining.

| **Secondary Antibody Name** | **Antibody Target** | **Host Species** | **Company & Catalogue Number** | **Dilution** |
| --- | --- | --- | --- | --- |
| Anti-Rabbit IgG (H+L) Highly Cross-Adsorbed Secondary Antibody, Alexa Fluor^TM^ Plus 647 | Polyclonal to Rabbit IgG (H+L) | Goat | Invitrogen^TM^, A32733 | 1:500 |
| Anti-Mouse IgG (H+L) Cross-Adsorbed Secondary Antibody, Alexa Fluor^TM^ 568 | Polyclonal to Mouse IgG (H+L) | Goat | Invitrogen^TM^, A-11004 | 1:500 |
| Anti-Goat IgG (H+L) Cross Adsorbed Secondary Antibody, Alexa Fluor^TM^ 546 | Polyclonal to Goat IgG (H+L) | Donkey | Invitrogen^TM^, A-11056 | 1:500 |

***Supplementary Table S3***: List of Conjugated Antibodies Used for Flow Cytometric Analyses.

| **Conjugated Antibody Name** | **Antibody Target** | **Host Species** | **Company & Catalogue Number** | **Dilution** |
| --- | --- | --- | --- | --- |
| V450 Anti-CD31 Antibody | Human CD31 | Mouse | BD Horizon^TM^, 561653 | 1:30 |
| FITC Anti-CD90 Antibody | Monoclonal to Human CD90 | Mouse | BIORAD, MCA90 | 1:100 |
| APC Anti-PDGF Receptor Alpha Antibody | Monoclonal to Human CD140a (PDGFRα) | Mouse | BioLegend®, 323512 | 1:50 |
| APC Anti-PDGF Receptor Beta Antibody | Monoclonal to Human CD140b (PDGFRβ) | Mouse | R&D Systems, FAB1263A | 1:50 |
| PE-CY7 Anti-CD45 Antibody | Monoclonal to Human CD45 (CD45) | Mouse | BioLegend®, 304016 | 1:50 |

***
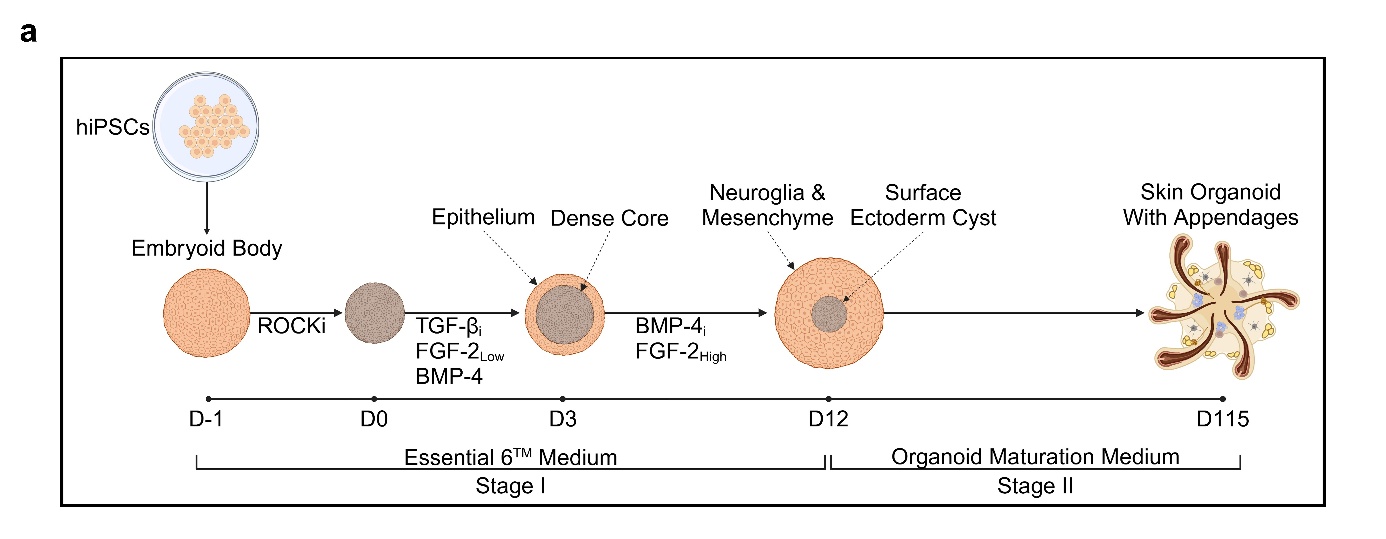
***

***Supplementary Figure S1: Generation of Human Skin Organoids Derived from Induced Pluripotent Stem Cells. a*** *Overview of the differentiation protocol to generate human induced pluripotent stem cell (hiPSC)-derived skin organoids (SKO). Created with BioRender.com. Bone morphogenetic protein 4 (BMP-4). Bone morphogenetic protein 4 inhibitor (BMP-4i). Fibroblast growth factor 2 (FGF-2). StemMACS^TM^ Y27632 (ROCKi, rho-associated kinase (ROCK) inhibitor). Transforming growth factor β inhibitor (TGF-β_i_).*
