# Supplementary material for: Coordinated Development of Immune Cell Populations in Vascularized Skin Organoids from Human Induced Pluripotent Stem Cells
Source: Adv Healthc Mater. 2025 Aug 16;14(31):e02108. doi: 10.1002/adhm.202502108 (PMC12683213; doi:10.1002/adhm.202502108)
Supplement: Supplementary file 2 — Supplementary Video1 [file ADHM-14-0-s002.pptx]

## Slide 1
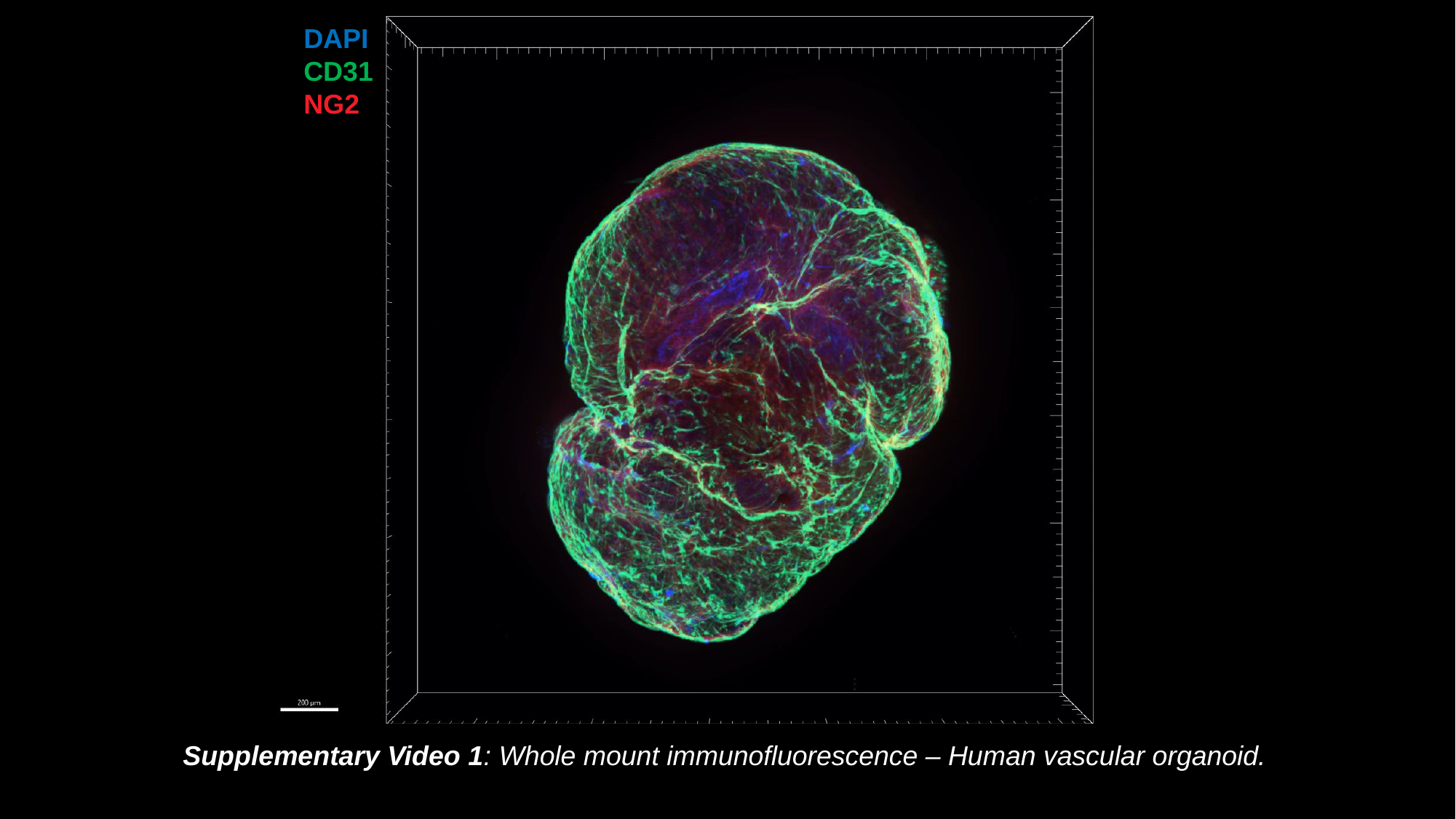

DAPI
CD31
NG2
Supplementary Video 1: Whole mount immunofluorescence – Human vascular organoid.
