# Supplementary material for: Coordinated Development of Immune Cell Populations in Vascularized Skin Organoids from Human Induced Pluripotent Stem Cells
Source: Adv Healthc Mater. 2025 Aug 16;14(31):e02108. doi: 10.1002/adhm.202502108 (PMC12683213; doi:10.1002/adhm.202502108)
Supplement: Supplementary file 4 — Supplementary Video3 [file ADHM-14-0-s004.pptx]

## Slide 1
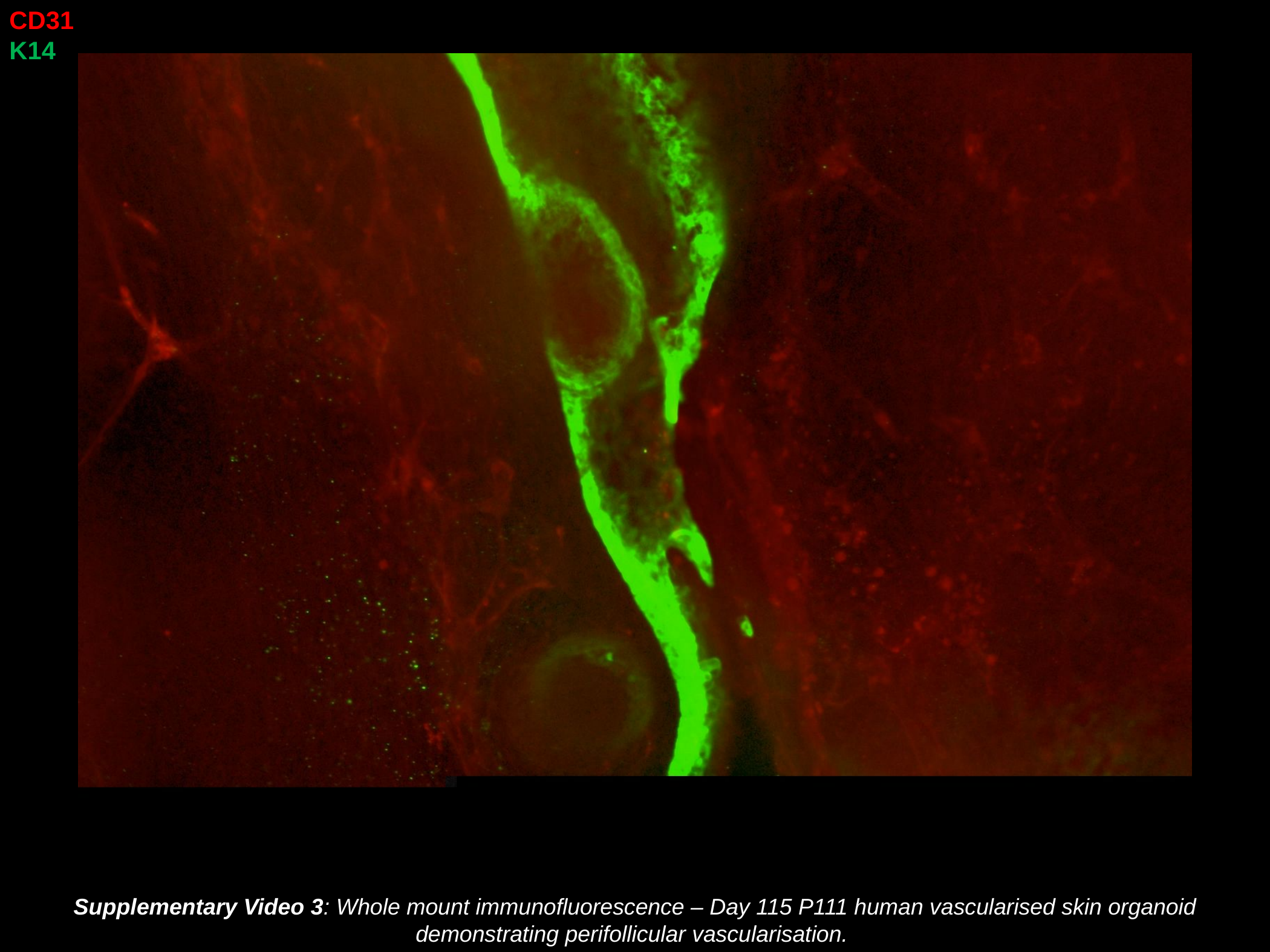

CD31
K14
Supplementary Video 3: Whole mount immunofluorescence – Day 115 P111 human vascularised skin organoid demonstrating perifollicular vascularisation.
